# Association of early lactate with 365-day all-cause mortality in critically ill patients with acute ischemic stroke: A retrospective cohort study using MIMIC-IV

**DOI:** 10.1097/MD.0000000000050184

**Published:** 2026-08-14

**Authors:** Yi Ba, Chengguang Song

**Affiliations:** a Second Ward, Department of Neurology, Benxi Central Hospital, Benxi, Liaoning, China.

**Keywords:** acute ischemic stroke, intensive care unit, lactate, MIMIC, mortality, prognosis

## Abstract

Lactate may integrate tissue hypoperfusion, adrenergic stress, impaired hepatic clearance, and metabolic responses to cerebral ischemia. Although not stroke-specific, early elevation may identify critically ill patients with concurrent neurological injury and extracerebral organ dysfunction. We evaluated the association between early lactate and all-cause mortality in critically ill patients with a hospitalization-level acute ischemic stroke (AIS) diagnosis. This retrospective cohort study used Medical Information Mart for Intensive Care IV version 3.1 (2008–2022). Adults with an AIS code in any diagnosis position were screened. The earliest lactate recorded during the index intensive care unit stay was used. The primary outcome was 365-day all-cause mortality. Adjusted Cox regression assessed time-to-event associations, and logistic regression evaluated fixed-horizon outcomes. Models were adjusted for demographics, comorbidity burden, neurological and global illness severity, organ dysfunction, hemodynamic and laboratory variables, and liver disease. Patients with and without lactate measurements were compared; calibration, restricted cubic splines, Kaplan–Meier curves, subgroup, decision curve, and sensitivity analyses were also performed. Among 2001 eligible patients, 859 had an early lactate measurement and 1142 did not; measured patients had greater illness severity and more frequent invasive ventilation. During 365 days, 286 patients (33.3%) died. Each 1 mmol/L lactate increase was associated with higher mortality (adjusted hazard ratio [HR], 1.14; 95% confidence interval [CI], 1.07–1.22; *P* < .001). Versus *Q*1, adjusted HRs were 1.74 (95% CI, 1.22–2.50) for *Q*3 and 2.00 (95% CI, 1.37–2.92) for *Q*4. Adding lactate increased the 5-fold cross-validated area under the curve from 0.744 to 0.755 and reduced the Brier score from 0.187 to 0.182, with calibration slopes of 0.87 and 0.86. Early lactate was associated with short- and long-term all-cause mortality in critically ill patients with a hospitalization-level AIS diagnosis. Selection into lactate testing was substantial, the incremental predictive gain was small, and external validation is required. Lactate should be interpreted as a complementary marker of systemic and neurological stress rather than a stand-alone predictor.

## 1. Introduction

Acute ischemic stroke remains a major cause of death and long-term disability worldwide. Although reperfusion therapy and organized stroke systems have improved early management, many patients still require intensive care because of impaired consciousness, respiratory failure, infection, hemodynamic instability, or other acute complications.^[1–3]^

Lactate is a routinely measured biomarker determined by both production and clearance. In critically ill patients, an elevated value may arise from systemic hypoperfusion, accelerated aerobic glycolysis driven by catecholamines, impaired hepatic clearance, seizures, infection, hypoxemia, or resuscitation. Its lack of stroke specificity, therefore, does not preclude prognostic relevance; rather, lactate may integrate several pathophysiological pathways that jointly influence recovery and survival.^[4,5]^

Acute cerebral ischemia can increase local glycolytic flux and tissue acidosis. Severe stroke may also precipitate impaired consciousness, aspiration, respiratory failure, autonomic dysregulation, and multiorgan dysfunction. A circulating lactate value cannot be interpreted as a direct measure of infarct volume, but it may provide an early summary signal of combined neurological injury, systemic perfusion deficit, metabolic stress, and limited physiological reserve.^[6–8]^

The Medical Information Mart for Intensive Care IV (MIMIC-IV) database contains detailed clinical, laboratory, treatment, and outcome data from patients admitted to the intensive care unit (ICU). Its deidentified structure and credentialed accessibility provide an opportunity to evaluate routinely measured biomarkers in large critical care cohorts.^[9,10]^

Accordingly, this study did not seek to establish lactate as a stroke-specific biomarker or to claim the discovery of an association already addressed by recent MIMIC-IV analyses. Instead, we examined whether the earliest routinely available lactate measurement remained associated with 30-, 90-, and 365-day all-cause mortality after accounting for measured neurological status, global illness severity, organ dysfunction, comorbidity burden, and liver disease. We also quantified selection into lactate testing, evaluated adjusted time-to-event and fixed-horizon models, and assessed the magnitude and clinical limits of any incremental predictive value.

## 2. Methods

### 2.1. Study design and data source

This retrospective cohort study used MIMIC-IV version 3.1, a credentialed-access, deidentified database derived from electronic health records at Beth Israel Deaconess Medical Center. Version 3.1 contains hospital and intensive care data collected from 2008 through 2022. The dataset used for this analysis was extracted on January 31, 2026 (export identifier 16502). Access to the source database required credentialing through PhysioNet, completion of the required human-subjects training, and acceptance of the data use agreement. MIMIC-IV was approved by the institutional review boards of Beth Israel Deaconess Medical Center and the Massachusetts Institute of Technology. Individual informed consent was waived because the data are deidentified. The present secondary analysis used only deidentified data in accordance with the PhysioNet data use agreement.

### 2.2. Study population

Adults aged 18 years or older were screened when an acute ischemic stroke (AIS) code was recorded during the hospitalization. The prespecified code set comprised International Classification of Diseases-9-CM 433.x1, 434.x1, and 436 and International Classification of Diseases-10-CM I63.x. A stroke code was not required to be the principal diagnosis; therefore, the primary cohort is described as critically ill patients with a hospitalization-level AIS diagnosis rather than patients admitted to the ICU exclusively because of stroke. For patients with multiple admissions or ICU stays, only the 1st hospital admission and 1st ICU stay were retained. Patients without a lactate result during that stay were excluded from the primary exposure analysis. A sensitivity analysis was restricted to patients whose 1st-listed diagnosis title contained cerebral infarction.

### 2.3. Exposure definition

The exposure was the earliest lactate result timestamped after ICU admission and before discharge from the 1st ICU stay; no fixed 24-hour upper limit was imposed. Lactate was analyzed as a continuous variable and by quartiles: *Q*1, ≤1.0 mmol/L; *Q2*, 1.01–1.4 mmol/L; *Q*3, 1.5–2.0 mmol/L; and *Q*4, ≥2.1 mmol/L. *Q*1 was the reference category.

### 2.4. Covariates

Variables were selected a priori on the basis of clinical relevance and database availability. Demographic information included age and sex. The Charlson comorbidity index is a weighted summary of chronic disease burden in which selected comorbidities contribute points according to their association with mortality; higher scores indicate a greater burden. Individual comorbidities were also described. Because hepatic clearance is central to lactate metabolism, mild or severe liver disease was combined into an any-liver-disease indicator and added to the fully adjusted association models. Alanine aminotransferase, aspartate aminotransferase, and total bilirubin were available in only 35.0%, 34.9%, and 33.6% of the lactate cohort, respectively, and were therefore not included in the primary complete-case model. Other covariates included Glasgow Coma Scale (GCS) score, Sequential Organ Failure Assessment (SOFA) score, sepsis, acute kidney injury (AKI), invasive ventilation, systolic blood pressure, heart rate, peripheral oxygen saturation (SpO2), creatinine, and glucose. The secondary predictive comparison retained the prespecified clinical model used in the original analysis, while liver disease was added to the association models requested by the reviewer.

### 2.5. Outcomes

The primary outcome was all-cause mortality within 365 days after ICU admission. This horizon was selected because the study focused on long-term survival, and MIMIC-IV provides linked out-of-hospital death information with follow-up censored at 1 year. The 30- and 90-day all-cause mortality outcomes were analyzed as secondary fixed horizons, with 90 days retained to facilitate comparison with conventional stroke studies. The modified Rankin Scale was not consistently available in MIMIC-IV. Cause-specific death was also unavailable; all-cause mortality was therefore used as the only objective and consistently captured mortality endpoint. Intensive care unit mortality, in-hospital mortality, and lengths of stay were also summarized.

### 2.6. Statistical analysis

Continuous variables are presented as medians with interquartile ranges and categorical variables as numbers with percentages. Baseline characteristics were compared across lactate quartiles using the Kruskal–Wallis and chi-square tests. To evaluate potential selection bias, patients with and without a lactate measurement were compared using the Mann–Whitney *U* test or chi-square test, as appropriate. For the primary 365-day time-to-event analysis, follow-up extended from ICU admission to death or administrative censoring at 365 days, and adjusted hazard ratios were estimated with Cox proportional hazards regression. Multivariable logistic regression was retained for the prespecified 30-, 90-, and 365-day fixed-horizon analyses. The fully adjusted association models included age, sex, Charlson comorbidity index, GCS score, SOFA score, sepsis, AKI, invasive ventilation, systolic blood pressure, heart rate, SpO_2_, creatinine, glucose, and any liver disease. Complete-case analysis was used because missingness in the primary covariates was low.

### 2.7. Additional analyses

Restricted cubic spline analysis was used to examine the continuous dose-response relation for 365-day mortality, and Kaplan–Meier curves were generated by lactate quartile. Five-fold cross-validated predictive performance was summarized by area under the receiver operating characteristic curve, Brier score, sensitivity, specificity, accuracy, positive predictive value, and negative predictive value. Calibration was further described by the calibration intercept and slope. The absolute change in area under the curve was accompanied by a paired bootstrap confidence interval and interpreted cautiously. Decision curve analysis assessed net benefit. Prespecified subgroup analyses used interaction terms. Sensitivity analyses excluded patients with sepsis, lactate values >10 mmol/L, and deaths within 24 hours, and, separately, restricted the cohort to a 1st-listed cerebral infarction diagnosis. All references to the mortality horizon were checked and standardized to 365 days.

### 2.8. Software

All statistical analyses were performed using Python version 3.13.5 (Python Software Foundation). Data management, statistical modeling, and visualization were completed using standard Python packages, including NumPy, SciPy, statsmodels, scikit-learn, and Matplotlib. All tests were 2-sided, and *P* < .05 was considered statistically significant.

## 3. Results

### 3.1. Patient selection and baseline characteristics

**Figure 1.**
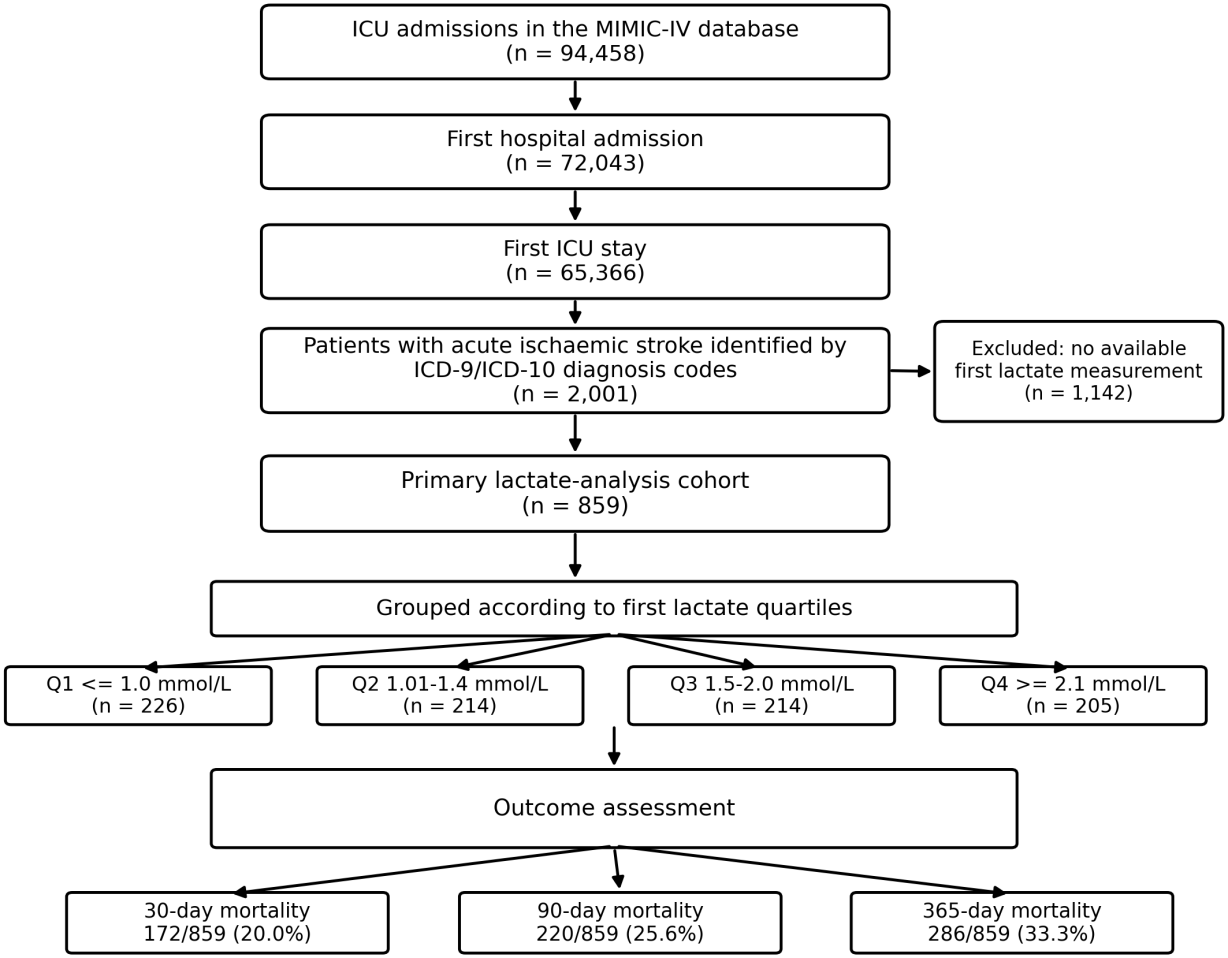
Flow diagram of patient selection. ICU = intensive care unit, ICD = International Classification of Diseases, MIMIC-IV = Medical Information Mart for Intensive Care IV.

A total of 2001 patients met the hospitalization-level AIS code definition after retention of the 1st admission and ICU stay. Of these, 859 had an early lactate measurement and 1142 did not (Fig. 1). The comparison between patients with and without lactate measurement is reported in Table S1, Supplemental Digital Content 1.

Compared with patients without lactate measurement, those with lactate measured had higher SOFA scores (median, 7 vs 3), higher acute physiology score III values (44 vs 35), more sepsis (73.8% vs 29.7%), more AKI (84.5% vs 60.4%), and more invasive ventilation (85.9% vs 27.0%); 365-day mortality was 33.3% versus 29.2%. These differences demonstrate clinically important selection into lactate testing. Within the lactate cohort, the median early lactate level was 1.4 mmol/L (interquartile range, 1.0–2.0), with 226, 214, 214, and 205 patients in *Q*1 through *Q*4, respectively. Baseline characteristics by quartile are shown in Table 1. Higher lactate quartiles were accompanied by lower GCS scores, higher SOFA and acute physiology score III values, and a higher prevalence of liver disease.

**Table 1:** Baseline characteristics according to 1st lactate quartiles.

Variable	Overall (n = 859)	*Q*1 (≤1.0) (n = 226)	*Q*2 (1.01–1.4) (n = 214)	*Q*3 (1.5–2.0) (n = 214)	*Q*4 (≥2.1) (n = 205)	*P* value
Age, yr	71.9 (61.7–80.3)	73.0 (64.6–82.0)	72.1 (63.8–78.9)	72.5 (62.7–81.1)	69.2 (58.0–79.1)	.011
Charlson comorbidity index	6 (4–8)	6 (5–8)	6 (5–8)	6 (4–8)	6 (4–8)	.244
GCS score	13 (7–14)	14 (9–15)	13 (8–14)	12 (8–14)	9 (4–14)	<.001
SOFA score	7 (5–9)	6 (4–8)	6 (4–8)	6 (5–9)	8 (6–11)	<.001
APS III score	44 (32–62)	40 (31–52)	40 (31–55)	42 (31–58)	57 (37–76)	<.001
Systolic blood pressure, mm Hg	122 (106–143)	120 (103–139)	126 (109–144)	122 (107–145)	121 (105–144)	.156
Heart rate, beats/min	82 (73–93)	80 (72–88)	80 (72–91)	82 (72–94)	88 (78–106)	<.001
SpO_2_, %	100 (97–100)	100 (99–100)	100 (97–100)	100 (98–100)	99 (96–100)	<.001
White blood cell count, ×10^9^/L	11.4 (8.2–15.5)	10.2 (7.3–13.5)	11.1 (8.1–14.9)	11.8 (9.3–16.1)	12.6 (9.1–18.6)	<.001
Creatinine, mg/dL	1.0 (0.7–1.3)	0.9 (0.7–1.2)	0.9 (0.8–1.3)	1.0 (0.8–1.3)	1.0 (0.8–1.6)	.005
Blood urea nitrogen, mg/dL	20.0 (14.0–28.0)	18.0 (14.0–25.0)	19.0 (14.0–26.2)	21.0 (15.0–29.0)	21.0 (14.0–30.5)	.033
Glucose, mg/dL	128 (104–168)	109 (96–138)	121 (104–156)	134 (108–172)	162 (126–215)	<.001
Lactate, mmol/L	1.4 (1.0–2.0)	0.8 (0.7–1.0)	1.2 (1.1–1.3)	1.7 (1.6–1.8)	2.9 (2.4–3.9)	<.001
Male sex	505 (58.8)	119 (52.7)	149 (69.6)	119 (55.6)	118 (57.6)	.002
Hypertension	484 (56.3)	123 (54.4)	137 (64.0)	107 (50.0)	117 (57.1)	.029
Diabetes mellitus	299 (34.8)	75 (33.2)	80 (37.4)	79 (36.9)	65 (31.7)	.537
Myocardial infarction	202 (23.5)	58 (25.7)	47 (22.0)	48 (22.4)	49 (23.9)	.795
Congestive heart failure	249 (29.0)	82 (36.3)	53 (24.8)	61 (28.5)	53 (25.9)	.034
Renal disease	186 (21.7)	53 (23.5)	43 (20.1)	49 (22.9)	41 (20.0)	.738
Chronic pulmonary disease	243 (28.3)	71 (31.4)	65 (30.4)	55 (25.7)	52 (25.4)	.374
Any liver disease	71 (8.3)	10 (4.4)	17 (7.9)	12 (5.6)	32 (15.6)	<.001
Severe liver disease	14 (1.6)	1 (0.4)	5 (2.3)	0 (0.0)	8 (3.9)	.005
Sepsis	634 (73.8)	156 (69.0)	156 (72.9)	158 (73.8)	164 (80.0)	.078
Acute kidney injury	726 (84.5)	187 (82.7)	181 (84.6)	185 (86.4)	173 (84.4)	.763
Invasive ventilation	738 (85.9)	194 (85.8)	176 (82.2)	185 (86.4)	183 (89.3)	.227

Values are shown as median (IQR) or n (%). *P* values were calculated using the Kruskal–Wallis test for continuous variables and the chi-square test for categorical variables. Any liver disease was defined as mild or severe liver disease.

APS III = Acute Physiology Score III, GCS = Glasgow Coma Scale, SOFA = Sequential Organ Failure Assessment, SpO_2_ = peripheral oxygen saturation.

### 3.2. Clinical outcomes across lactate quartiles

Clinical outcomes are summarized in Table 2. Hospital length of stay was similar across quartiles, whereas ICU stay became longer with increasing lactate. The median ICU stay was 3.0 days in *Q*1 and 5.8 days in *Q*4 (*P* < .001). Mortality increased stepwise across lactate quartiles. Intensive care unit mortality rose from 6.6% in *Q*1 to 21.5% in *Q*4, and in-hospital mortality rose from 8.0% to 30.2% (both *P* < .001). The same pattern was observed for 28-, 30-, 90-, and 365-day mortality. The 365-day mortality rate increased from 51 of 226 patients (22.6%) in *Q*1 to 102 of 205 patients (49.8%) in *Q*4 (*P* < .001).

**Table 2:** Clinical outcomes according to 1st lactate quartiles.

Variable	Overall (n = 859)	*Q*1 (≤1.0) (n = 226)	*Q*2 (1.01–1.4) (n = 214)	*Q*3 (1.5–2.0) (n = 214)	*Q*4 (≥2.1) (n = 205)	*P* value
Length of hospital stay, days	10.0 (6.3–16.7)	10.0 (6.5–14.7)	9.7 (6.5–15.9)	9.3 (6.0–15.5)	11.6 (6.4–19.7)	.371
Length of ICU stay, days	3.4 (2.0–7.9)	3.0 (1.5–5.6)	3.2 (2.0–6.4)	3.6 (2.1–8.1)	5.8 (2.2–9.4)	<.001
ICU mortality	102 (11.9)	15 (6.6)	19 (8.9)	24 (11.2)	44 (21.5)	<.001
In-hospital mortality	151 (17.6)	18 (8.0)	28 (13.1)	43 (20.1)	62 (30.2)	<.001
28-d mortality	172 (20.0)	23 (10.2)	28 (13.1)	51 (23.8)	70 (34.1)	<.001
30-d mortality	172 (20.0)	23 (10.2)	28 (13.1)	51 (23.8)	70 (34.1)	<.001
90-d mortality	220 (25.6)	29 (12.8)	39 (18.2)	63 (29.4)	89 (43.4)	<.001
365-d mortality	286 (33.3)	51 (22.6)	57 (26.6)	76 (35.5)	102 (49.8)	<.001

Values are shown as median (IQR) or n (%).

ICU = intensive care unit.

### 3.3. Association between 1st lactate level and 365-day mortality

In the primary adjusted Cox analysis of 849 complete cases with 280 deaths, each 1 mmol/L increase in early lactate was associated with a 14% higher hazard of death within 365 days (adjusted hazard ratio [HR], 1.14; 95% confidence interval [CI], 1.07–1.22; *P* < .001). Compared with *Q*1, adjusted HRs were 1.08 (95% CI, 0.73–1.59) for *Q*2, 1.74 (95% CI, 1.22–2.50) for *Q*3, and 2.00 (95% CI, 1.37–2.92) for *Q*4. The model adjusted for age, sex, Charlson comorbidity index, GCS score, SOFA score, sepsis, AKI, invasive ventilation, systolic blood pressure, heart rate, SpO_2_, creatinine, glucose, and any liver disease. The corresponding fixed-horizon logistic model was concordant (adjusted odds ratio [OR] per 1 mmol/L, 1.28; 95% CI, 1.13–1.46; *P* < .001) (Table 3).

**Table 3:** Adjusted association between early lactate and 365-day all-cause mortality.

Analysis	Lactate term	Effect	Lower 95% CI	Upper 95% CI	*P* value
Adjusted Cox	Per 1 mmol/L increase	HR 1.14	1.07	1.22	<.001
Adjusted Cox	*Q*2 vs *Q*1	HR 1.08	0.73	1.59	.714
Adjusted Cox	*Q*3 vs *Q*1	HR 1.74	1.22	2.50	.002
Adjusted Cox	*Q*4 vs *Q*1	HR 2.00	1.37	2.92	<.001
Adjusted logistic	Per 1 mmol/L increase	OR 1.28	1.13	1.46	<.001
Adjusted logistic	*Q*2 vs *Q*1	OR 1.10	0.68	1.78	.695
Adjusted logistic	*Q*3 vs *Q*1	OR 1.62	1.02	2.57	.040
Adjusted logistic	*Q*4 vs *Q*1	OR 2.35	1.43	3.86	.001

Cox and logistic models were adjusted for age, sex, Charlson comorbidity index, Glasgow Coma Scale score, Sequential Organ Failure Assessment score, sepsis, acute kidney injury, invasive ventilation, systolic blood pressure, heart rate, peripheral oxygen saturation, creatinine, glucose, and any liver disease. Cox follow-up extended from intensive care unit admission to death or censoring at 365 days.

CI = confidence interval, HR = hazard ratio, OR = odds ratio.

**Figure 2.**
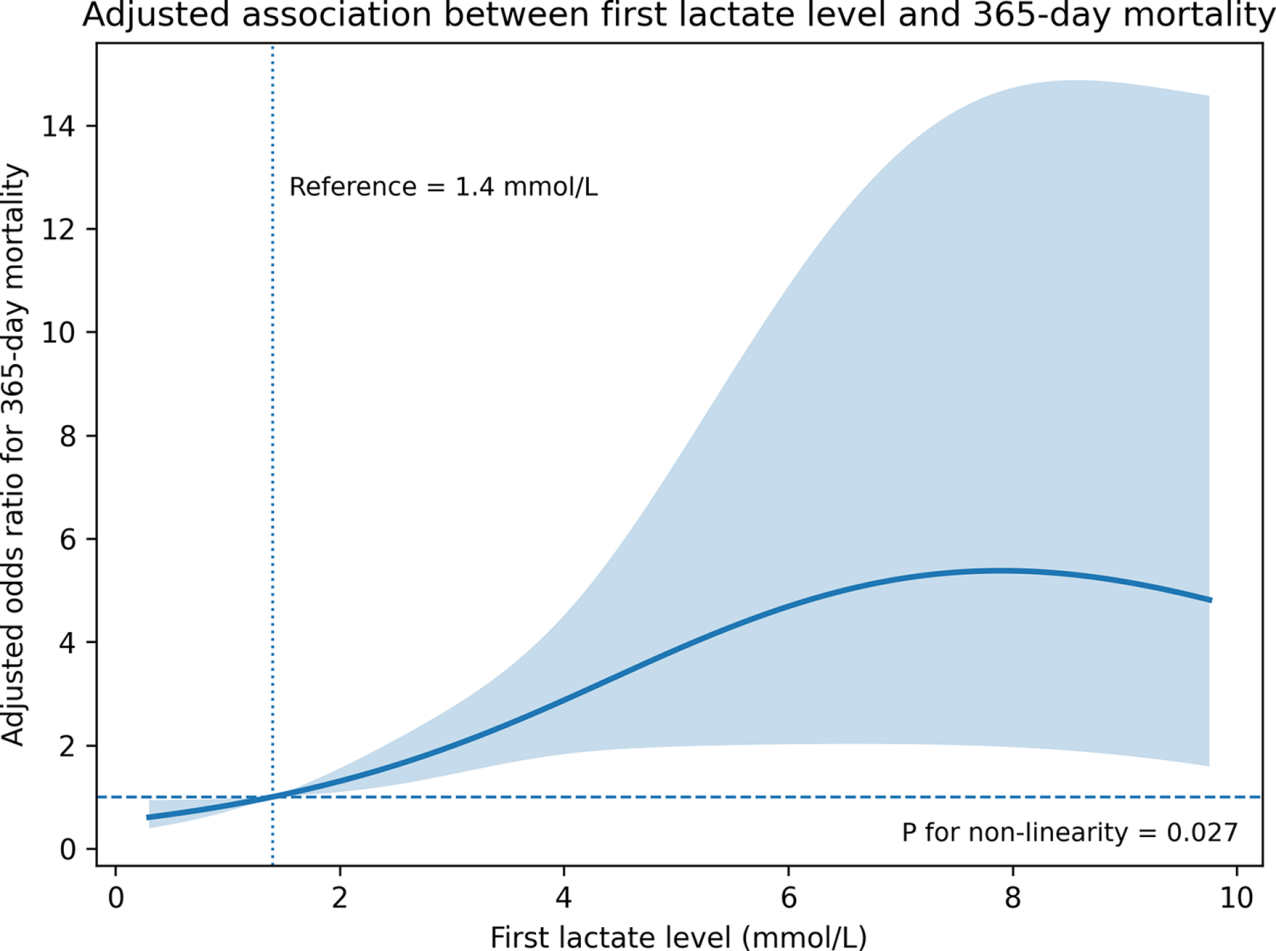
Restricted cubic spline analysis of the adjusted association between 1st lactate level and 365-day mortality. The curve shows the adjusted odds ratio, and the shaded area represents the 95% confidence interval.

**Figure 3.**
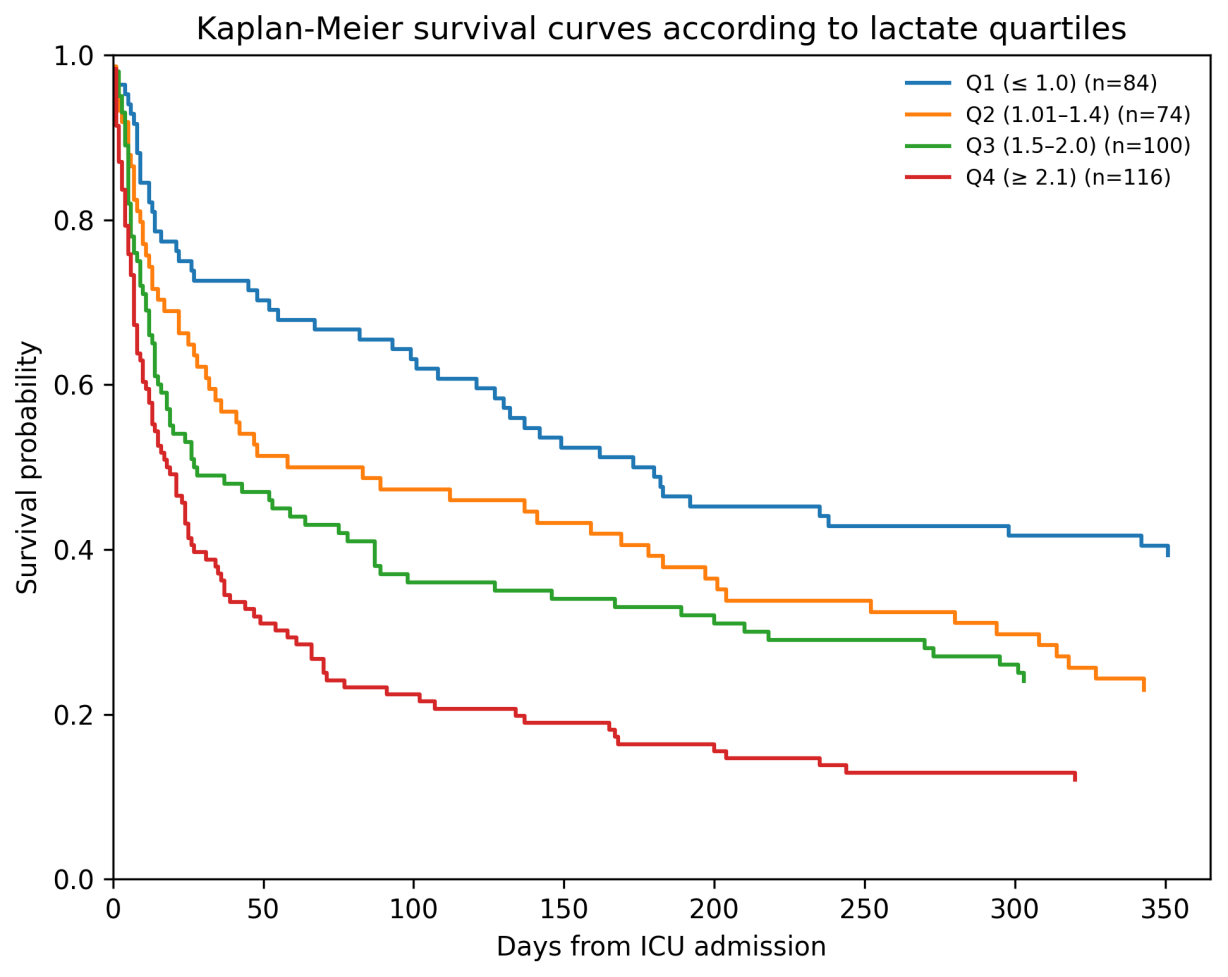
Kaplan–Meier survival curves for 365-day mortality according to 1st lactate quartiles. ICU = intensive care unit.

Restricted cubic spline analysis supported a positive adjusted association between early lactate and 365-day mortality (Fig. 2). Risk increased as lactate rose above the reference level of 1.4 mmol/L, with evidence of nonlinearity (*P* for nonlinearity = .027). Kaplan–Meier analysis also showed progressively lower survival probability with increasing lactate quartiles during follow-up (Fig. 3).

### 3.4. Predictive performance for 365-day mortality

**Figure 4.**
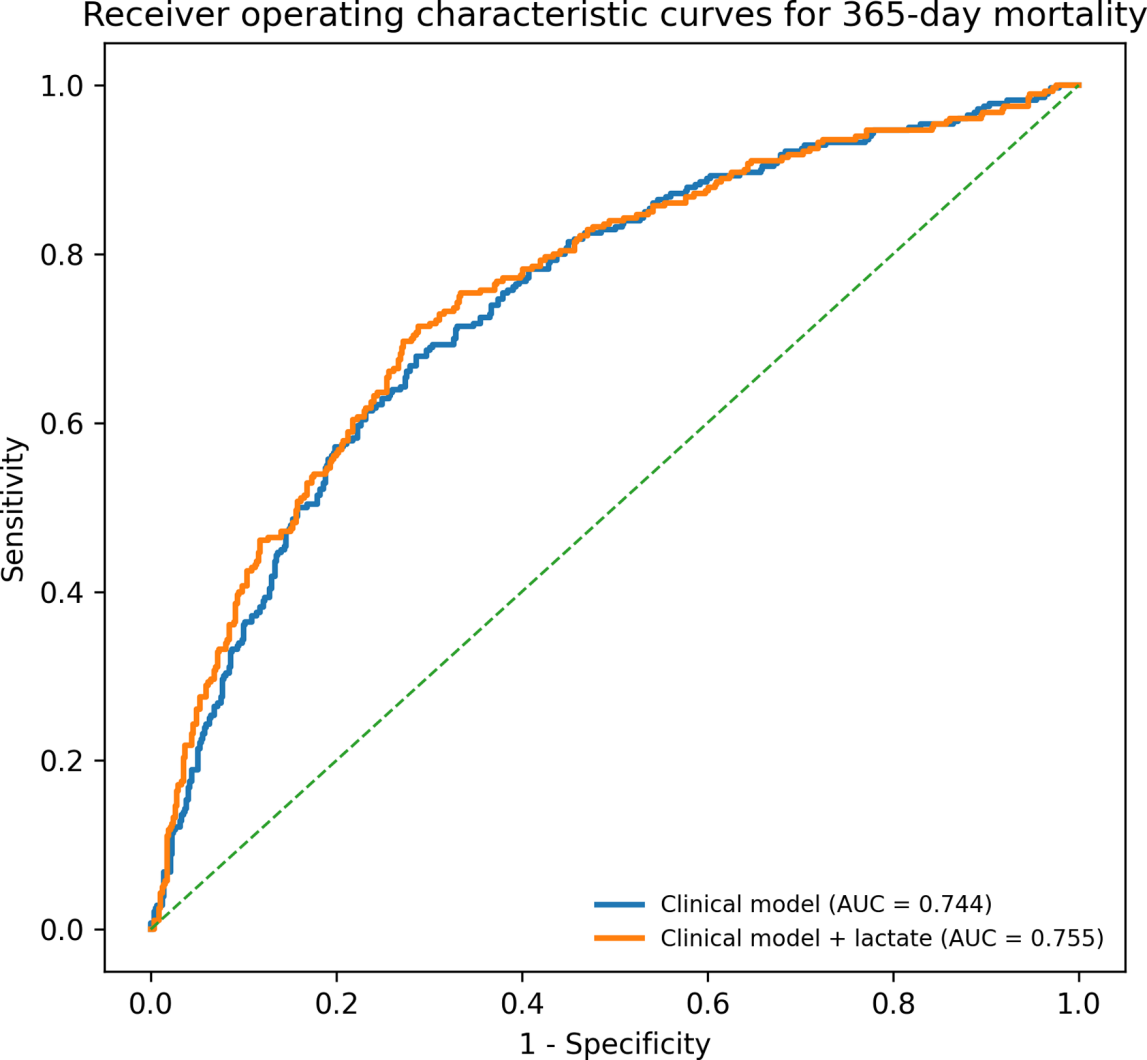
Receiver operating characteristic curves comparing the prespecified clinical model with and without early lactate for 5-fold cross-validated prediction of 365-day mortality. AUC = area under the receiver operating characteristic curve.

The prespecified clinical model had a 5-fold cross-validated area under the curve (AUC) of 0.744, and the lactate-added model had an AUC of 0.755 (absolute increase, 0.011; paired bootstrap 95% CI, 0.001–0.023) (Fig. 4 and Table 4). The Brier score decreased from 0.187 to 0.182. Calibration intercepts were approximately −0.08 for both models, and calibration slopes were 0.87 and 0.86, respectively. These results indicate a small improvement in discrimination and overall prediction error, without evidence that lactate materially transforms model performance.

**Table 4:** Cross-validated discrimination and calibration for 365-day mortality.

Model	N	Events	AUC	AUC change	Brier score	Calibration intercept	Calibration slope
Clinical model	849	280	0.744	Reference	0.187	−0.08	0.87
Clinical model + lactate	849	280	0.755	0.011 (95% CI, 0.001–0.023)	0.182	−0.08	0.86

Out-of-fold predictions were generated using 5-fold stratified cross-validation. The clinical prediction model retained the prespecified variables from the original analysis; lactate was then added as a single additional predictor. The confidence interval for the AUC change was obtained by paired bootstrap resampling of out-of-fold predictions.

AUC = area under the receiver operating characteristic curve.

### 3.5. Short- and intermediate-term mortality

The fixed-horizon association was also present for earlier outcomes after adjustment for any liver disease and the other prespecified covariates (Table 5). Each 1 mmol/L increase in lactate was associated with 30-day mortality (adjusted OR, 1.26; 95% CI, 1.11–1.44; *P* < .001) and 90-day mortality (adjusted OR, 1.33; 95% CI, 1.16–1.53; *P* < .001). Compared with *Q*1, *Q*3, and *Q*4 were associated with higher mortality at both horizons, whereas *Q*2 was not significantly different.

**Table 5:** Adjusted fixed-horizon logistic regression analyses.

Outcome	N	Events	Lactate term	Adjusted OR	Lower 95% CI	Upper 95% CI	*P* value
30-d	849	168	Per 1 mmol/L	1.26	1.11	1.44	<.001
30-d	849	168	*Q*2 vs *Q*1	1.14	0.61	2.16	.676
30-d	849	168	*Q*3 vs *Q*1	2.34	1.32	4.14	.004
30-d	849	168	*Q*4 vs *Q*1	2.79	1.53	5.07	.001
90-d	849	215	Per 1 mmol/L	1.33	1.16	1.53	<.001
90-d	849	215	*Q*2 vs *Q*1	1.30	0.73	2.30	.368
90-d	849	215	*Q*3 vs *Q*1	2.45	1.44	4.16	.001
90-d	849	215	*Q*4 vs *Q*1	3.27	1.87	5.72	<.001

Models were adjusted for age, sex, Charlson comorbidity index, Glasgow Coma Scale score, Sequential Organ Failure Assessment score, sepsis, acute kidney injury, invasive ventilation, systolic blood pressure, heart rate, peripheral oxygen saturation, creatinine, glucose, and any liver disease.

CI = confidence interval, OR = odds ratio.

### 3.6. Incremental predictive performance across mortality horizons

Incremental predictive performance across mortality horizons is summarized in Table 6. The absolute AUC increases after adding lactate were small: approximately 0.014 for 30-day mortality, 0.016 for 90-day mortality, and 0.011 for 365-day mortality. Brier scores improved slightly, and calibration slopes remained below 1 in both the clinical and lactate-added models. Accordingly, these analyses support complementary rather than stand-alone prognostic use.

**Table 6:** Cross-validated performance across mortality horizons.

Outcome	Model	AUC	Brier	Calibration intercept	Calibration slope
30-d	Clinical	0.749	0.140	−0.22	0.82
30-d	Clinical + lactate	0.763	0.136	−0.19	0.84
90-d	Clinical	0.761	0.162	−0.19	0.80
90-d	Clinical + lactate	0.777	0.155	−0.18	0.81
365-d	Clinical	0.744	0.187	−0.08	0.87
365-d	Clinical + lactate	0.755	0.182	−0.08	0.86

Out-of-fold predictions were generated using 5-fold stratified cross-validation. The clinical prediction model retained the prespecified variables from the original analysis, and lactate was added as 1 additional predictor.

AUC = area under the receiver operating characteristic curve.

### 3.7. Subgroup, decision curve, and sensitivity analyses

**Figure 5.**
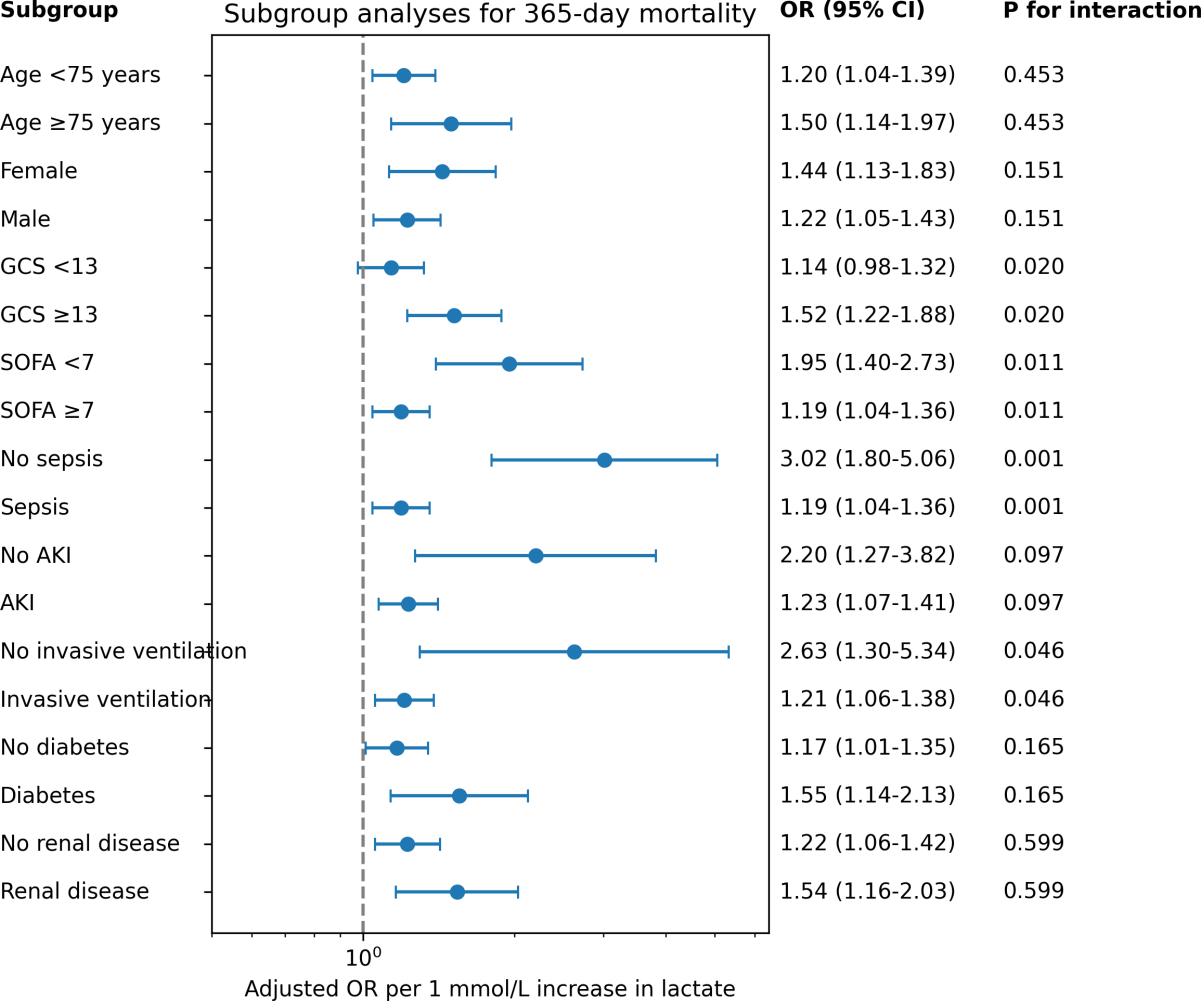
Subgroup analysis of the association between 1st lactate level and 365-day mortality. Odds ratios were estimated within prespecified clinical subgroups. AKI = acute kidney injury, CI = confidence interval, GCS = Glasgow Coma Scale, OR = odds ratio, SOFA = Sequential Organ Failure Assessment.

Subgroup analyses are shown in Figure 5. The direction of the association between early lactate and 365-day mortality was generally consistent across most clinically relevant subgroups, including age, sex, AKI, diabetes, and renal disease. The magnitude of the association varied across several strata, with statistically significant interaction tests for GCS category, SOFA category, sepsis status, and invasive ventilation status. These findings suggest that the prognostic signal of lactate may depend on baseline neurological status and systemic critical illness.

**Figure 6.**
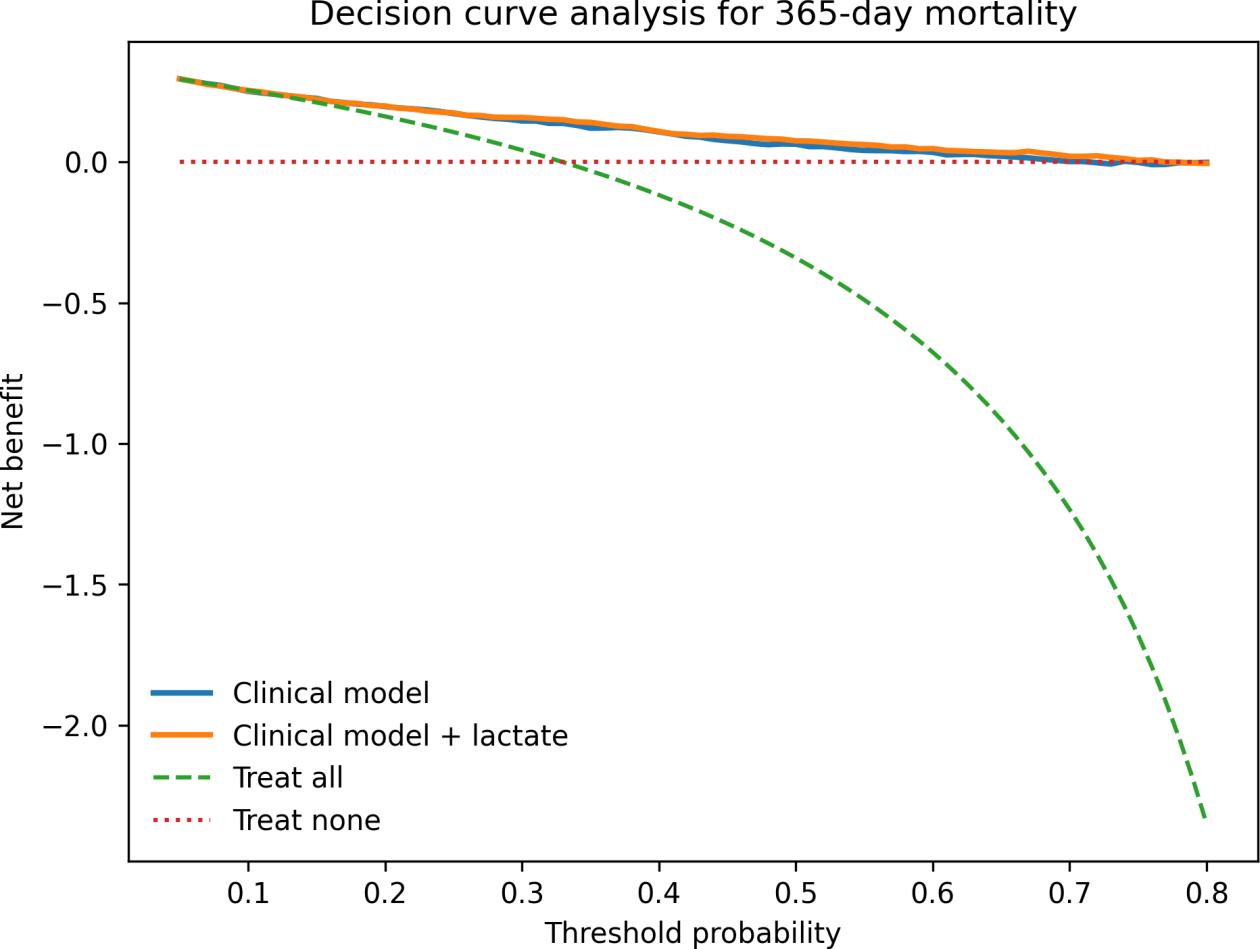
Decision curve analysis comparing the clinical model with the lactate-added model for prediction of 365-day mortality.

Decision curve analysis showed only a slight net-benefit advantage for the lactate-added model across part of the threshold range (Fig. 6). Corrected sensitivity analyses are reported in Table S2, Supplemental Digital Content 2. The adjusted OR per 1 mmol/L was 2.94 (95% CI, 1.75–4.92) after excluding sepsis; this larger estimate arose in a much smaller subgroup (n = 222) and should be interpreted cautiously. The association persisted after excluding lactate values >10 mmol/L (OR, 1.40; 95% CI, 1.21–1.62) and after correctly excluding deaths within 24 hours (n = 836; OR, 1.23; 95% CI, 1.08–1.40). In patients with a 1st-listed cerebral infarction diagnosis, the association also remained present (n = 125; adjusted Cox HR, 1.66; 95% CI, 1.21–2.29).

## 4. Discussion

### 4.1. Principal findings

In this retrospective cohort, early lactate was associated with 30-, 90-, and 365-day all-cause mortality after adjustment for measured neurological status, global illness severity, organ dysfunction, comorbidity burden, and liver disease. The adjusted Cox analysis showed that the 365-day association was not an artifact of treating the outcome as a simple binary endpoint. At the same time, the included-versus-excluded comparison showed that lactate was preferentially measured in substantially sicker patients, and the improvement in prediction was modest. The findings, therefore, support lactate as an integrative severity marker, not as a specific surrogate for infarct size or a causal determinant of outcome.

### 4.2. Comparison with previous studies

The present study substantially overlaps with the 2025 MIMIC-IV report by Zhao and colleagues and with other studies of lactate-related indices in ischemic stroke. We, therefore, do not claim discovery of a previously unknown association. The contribution of the current analysis is narrower: it quantifies selection into lactate testing, adds hepatic comorbidity to the adjusted association models, uses an adjusted time-to-event model for 365-day mortality, reports calibration in addition to discrimination, corrects the early-death sensitivity analysis, and tests the association in a 1st-listed cerebral infarction subgroup. These refinements help define the robustness and practical limits of a single early lactate measurement. The increasingly crowded MIMIC-IV biomarker literature also suggests that the clinically relevant question is not whether another routine variable is statistically associated with mortality, but whether it adds reproducible and meaningful information beyond established severity indicators.^[11–24]^

### 4.3. Possible mechanisms

Several mechanisms may underlie the association. Cerebral ischemia increases glycolysis and tissue acidosis locally, but circulating lactate in an intensive care population also reflects a systemic oxygen delivery imbalance, beta-adrenergic stimulation, infection, respiratory failure, seizures, renal dysfunction, hepatic clearance, and resuscitation. Larger or more severe strokes may contribute through reduced consciousness, aspiration, neurogenic cardiopulmonary complications, and autonomic stress; however, lactate cannot distinguish these pathways. The persistence of the association after adjustment for GCS and organ failure scores suggests that lactate carries information not fully captured by those variables, but residual confounding by infarct volume, National Institutes of Health Stroke Scale score, reperfusion treatment, and treatment timing remains likely.

### 4.4. Clinical implications

Lactate is inexpensive and rapidly available, but the observed AUC gain was only 0.011 for 365-day mortality, and calibration was not materially improved. It should therefore not trigger treatment decisions in isolation. Its most defensible role is as a prompt to reassess perfusion, oxygenation, infection, seizure activity, liver function, and evolving organ dysfunction while integrating neurological examination, hemodynamic measures, comorbidities, and treatment factors. This interpretation is consistent with contemporary prediction model guidance emphasizing calibration, clinical utility, and external validation rather than reliance on a single marker.^[25,26]^ The broader critical care literature similarly treats lactate as a marker of perfusion and metabolic stress. In cardiogenic shock complicating ST-segment elevation myocardial infarction and in acute coronary syndrome requiring intra-aortic balloon pump support, elevated lactate identified patients with unfavorable in-hospital trajectories.^[27,28]^ A recent multivariable risk model study in older nonelective surgical patients likewise illustrates that biomarkers are most useful when integrated with clinical and organ function variables and validated for the intended setting.^[29]^

### 4.5. Subgroup and sensitivity analyses

The sensitivity analyses reinforce the need for clinical context. The larger estimate in patients without sepsis was independently verified, but that subgroup was small and susceptible to instability. The corrected early death analysis retained statistical significance, indicating that the main association was not driven solely by patients who died immediately after admission. Nevertheless, significant interactions with neurological and systemic severity measures indicate that lactate does not have a uniform meaning across all critically ill patients.

### 4.6. Strengths and limitations

This study has several strengths, including complementary time-to-event and fixed-horizon analyses, explicit assessment of selection into lactate testing, adjustment for liver disease, evaluation of calibration and decision curves, and sensitivity analyses based on diagnosis position. Important limitations remain. First, the observational design supports association but not causality. Second, only 859 of 2001 eligible patients had lactate measured, and the comparison with excluded patients demonstrated substantial selection related to critical illness. Third, the stroke code was not required to be the principal diagnosis; although a 1st-listed cerebral infarction sensitivity analysis was consistent, the primary cohort may include patients admitted mainly for concurrent conditions. Fourth, the cause of death was unavailable, so deaths unrelated to stroke may dilute or confound the relation between early lactate and stroke prognosis. Fifth, the National Institutes of Health Stroke Scale score, infarct volume, vascular imaging, reperfusion status, seizures, and treatment timing were not consistently available.^[30–33]^ Sixth, aminotransferases and bilirubin were too incomplete for primary adjustment; liver disease coding reduces but does not eliminate confounding from impaired clearance. Finally, the single-center database and internal validation limit generalizability; independent external validation is essential before clinical implementation.

## 5. Conclusion

In critically ill patients with a hospitalization-level AIS diagnosis, higher early lactate was associated with increased 30-, 90-, and 365-day all-cause mortality after adjustment for measured confounders, including liver disease. Selection into lactate testing was substantial, and the incremental predictive gain was small. Lactate should be interpreted as 1 component of a broader assessment of neurological and systemic illness rather than as a stand-alone predictor. Independent external validation is necessary before lactate-based risk assessment is used for routine clinical decisions.

## Author contributions

**Conceptualization:** Yi Ba.

**Data curation:** Yi Ba.

**Formal analysis:** Yi Ba.

**Funding acquisition:** Yi Ba.

**Investigation:** Yi Ba.

**Methodology:** Yi Ba.

**Project administration:** Yi Ba.

**Resources:** Yi Ba, Chengguang Song.

**Software:** Yi Ba, Chengguang Song.

**Supervision:** Yi Ba, Chengguang Song.

**Validation:** Yi Ba, Chengguang Song.

**Visualization:** Yi Ba, Chengguang Song.

**Writing – original draft:** Yi Ba, Chengguang Song.

**Writing – review & editing:** Yi Ba, Chengguang Song.




