# Supplementary material for: Association of early lactate with 365-day all-cause mortality in critically ill patients with acute ischemic stroke: A retrospective cohort study using MIMIC-IV
Source: Medicine (Baltimore). 2026 Aug 14;105(33):e50184. doi: 10.1097/MD.0000000000050184 (PMC13480853; doi:10.1097/MD.0000000000050184)
Supplement: Supplementary file 1 [file medi-105-e50184-s001.docx]

**Supplementary Table 1. Comparison of eligible patients with and without lactate measurement**

| **Variable** | **Lactate available (n=859)** | **Lactate unavailable (n=1142)** | **P-value** |
| --- | --- | --- | --- |
| Age, years | 71.9 (61.7-80.3) | 73.2 (61.6-82.9) | 0.027 |
| Male sex | 505 (58.8) | 564 (49.4) | <0.001 |
| Charlson comorbidity index | 6 (4-8) | 6 (4-8) | 0.673 |
| GCS score | 13 (7-14) | 13 (9-15) | <0.001 |
| SOFA score | 7 (5-9) | 3 (2-5) | <0.001 |
| APS III score | 44 (32-61.5) | 35 (28-47) | <0.001 |
| Systolic blood pressure, mmHg | 122 (106-143) | 141 (124-158) | <0.001 |
| Heart rate, beats/min | 82 (73-93) | 79 (69-92) | <0.001 |
| SpO2, % | 100 (97-100) | 97 (95-100) | <0.001 |
| Creatinine, mg/dL | 1.0 (0.7-1.3) | 0.9 (0.7-1.2) | 0.002 |
| Glucose, mg/dL | 128 (104-168) | 124 (104-153) | 0.001 |
| Sepsis | 634 (73.8) | 339 (29.7) | <0.001 |
| Acute kidney injury | 726 (84.5) | 690 (60.4) | <0.001 |
| Invasive ventilation | 738 (85.9) | 308 (27.0) | <0.001 |
| Any liver disease | 71 (8.3) | 45 (3.9) | <0.001 |
| ICU length of stay, days | 3.4 (2.0-7.9) | 1.9 (1.1-3.6) | <0.001 |
| Hospital length of stay, days | 10.0 (6.3-16.7) | 6.4 (3.6-11.9) | <0.001 |
| 30-day mortality | 172 (20.0) | 215 (18.8) | 0.502 |
| 90-day mortality | 220 (25.6) | 267 (23.4) | 0.250 |
| 365-day mortality | 286 (33.3) | 333 (29.2) | 0.048 |

Values are shown as median (IQR) or n (%). Continuous variables were compared with the Mann-Whitney U test and categorical variables with the chi-square test. Any liver disease was defined as mild or severe liver disease. APS III, Acute Physiology Score III; GCS, Glasgow Coma Scale; ICU, intensive care unit; SOFA, Sequential Organ Failure Assessment; SpO2, peripheral oxygen saturation.
