# Supplementary material for: Association of early lactate with 365-day all-cause mortality in critically ill patients with acute ischemic stroke: A retrospective cohort study using MIMIC-IV
Source: Medicine (Baltimore). 2026 Aug 14;105(33):e50184. doi: 10.1097/MD.0000000000050184 (PMC13480853; doi:10.1097/MD.0000000000050184)
Supplement: Supplementary file 2 [file medi-105-e50184-s002.docx]

**Supplementary Table 2. Sensitivity analyses for 365-day mortality**

| **Analysis** | **N** | **Events** | **Adjusted estimate per 1 mmol/L** | **Lower 95% CI** | **Upper 95% CI** | **P-value** |
| --- | --- | --- | --- | --- | --- | --- |
| Primary logistic analysis | 849 | 280 | OR 1.28 | 1.13 | 1.46 | <0.001 |
| Excluding sepsis | 222 | 55 | OR 2.94 | 1.75 | 4.92 | <0.001 |
| Excluding lactate >10 mmol/L | 845 | 278 | OR 1.40 | 1.21 | 1.62 | <0.001 |
| Excluding deaths within 24 h | 836 | 267 | OR 1.23 | 1.08 | 1.40 | 0.002 |
| First-listed cerebral infarction diagnosis | 125 | 65 | Cox HR 1.66 | 1.21 | 2.29 | 0.002 |

The primary and sensitivity logistic models were adjusted for the same covariates as the main association model; a covariate that became constant within a restricted subgroup was omitted from that subgroup model. The first-listed cerebral infarction analysis used adjusted Cox regression. CI, confidence interval; HR, hazard ratio; OR, odds ratio.
